# Evaluation of a HIV Voluntary Opt-Out Screening Program in a Singapore Hospital

**DOI:** 10.1371/journal.pone.0116987

**Published:** 2015-01-22

**Authors:** Xin Quan Tan, Wei-Ping Goh, Indumathi Venkatachalam, Diana Goh, Revathi Sridhar, Hwang Ching Chan, Sophia Archuleta

**Affiliations:** 1 Division of Infectious Diseases, University Medicine Cluster, National University Hospital Singapore, Singapore, Singapore; 2 Division of General Medicine and Therapeutics, Department of Medicine, National University Hospital Singapore, Singapore, Singapore; 3 Epidemiology Unit, Division of Infectious Diseases, Department of Medicine, National University Hospital Singapore, Singapore, Singapore; 4 Department of Medicine, Yong Loo Lin School of Medicine, National University of Singapore, Singapore, Singapore; University of Missouri-Kansas City, UNITED STATES

## Abstract

**Background:**

Early diagnosis of human immunodeficiency virus (HIV) allows for appropriately timed interventions with improved outcomes, but HIV screening among asymptomatic persons and the general population in Singapore remains low. In 2008, Singapore’s Ministry of Health implemented HIV voluntary opt-out screening (VOS) for hospitalised adults. We evaluated the outcome of VOS and surveyed reasons for its low uptake in our institution.

**Methods:**

We assessed the outcomes of the VOS programme from January 2010 to December 2013 at National University Hospital, a 1081-bed tertiary hospital in Singapore. We also examined reasons for opting-in and opting-out using an interviewer–administered structured questionnaire in a representative sample in January 2013.

**Results:**

107,523 patients fulfilled VOS criteria and were offered HIV screening, of which 5215 (4.9%) agreed to testing. 4850 (93.1%) of those who opted-in had an HIV test done. Three (0.06%) tested positive for HIV. 238 patients (14.2%) were surveyed regarding reasons for opting-in or out of VOS. 21 (8.8%) had opted-in. Patients who opted-in were likely to be younger, more educated and reported having more regular sexual partners. Type of housing, number of casual sexual partners, sexual orientation, intravenous drug use, condom use and previous sexually transmitted infection were not associated with deciding to opt-in/out. Patients’ most common reasons for opting-out were: belief that they were at low risk (50.2%), belief that they were too old (26.8%), cost (6.9%) and aversion to venepuncture (6.5%). The most common reason for opting-in was desire to know their HIV status (47.6%).

**Conclusion:**

The success of an HIV-VOS program is largely determined by test uptake. Our study showed that the majority of eligible VOS patients opted-out of HIV screening. Given the considerable cost and low yield of this programme, more needs to be done to better equip patients in self-risk assessment and opting in to testing.

## Introduction

As of 31 Dec 2013, 6,229 persons have received a diagnosis of human immunodeficiency virus (HIV) infection in Singapore, and 1,671 (28.1%) have died. Of the 454 new HIV cases reported among Singapore residents in 2013, 41% had late stage infection [1]. Early diagnosis of HIV allows for appropriately timed interventions, which can lead to reduced viral transmission as well as improved health outcomes, including slower clinical progression, reduced risk of transmission to uninfected partners, and reduced mortality [2–4]. However, HIV continues to be diagnosed at a late stage in Singapore while uptake of screening among asymptomatic persons and the general population remains low.

The United States Centers for Disease Control and Prevention issued recommendations in 2006 for health-care providers to initiate HIV screening in regions where the prevalence of undiagnosed HIV is ≥0.1% [5]. A seroprevalence study conducted in five public hospitals in Singapore estimated the undiagnosed HIV prevalence to be 0.28% [6]. This led Singapore’s Ministry of Health (MOH) to implement a HIV voluntary opt-out screening (VOS) programme for all adult public hospital inpatients. At a systems level, based on the seroprevalence of undiagnosed HIV in public hospital admissions, up to 800 undiagnosed cases could potentially be picked up through this VOS programme in a year.

The VOS programme began in 2008 and is still ongoing with an aim to reduce the prevalence of undiagnosed and late stage diagnosis of HIV. This study evaluated the outcomes of the VOS programme and surveyed reasons for its low uptake [7] in our institution.

## Methods

### Site

The study was conducted in National University Hospital (NUH), a 1081-bed public tertiary care hospital in Singapore, with active haematology, oncology, neurosurgery, cardiothoracic surgery and transplant services covering both pediatrics and adults. During the 48-month study period of January 2010 to December 2013, there were 231,227 admissions accounting for 1,309,659 patient-days and (57,807 admissions/year, caseload 327,415 patient-days/year).

### VOS Programme

All inpatients ≥ 21 years were eligible for the VOS programme unless the following exclusion criteria were met: unable to opt-out due to mental incapacity or language barriers, screened for HIV within the past one year, admitted under services with existing HIV screening programmes (i.e. cardiothoracic, obstetrics, transplant, or haemodialysis wards), or admitted to the intensive care or high dependency units. Our analysis excluded patients with clinical reason to suspect HIV infection (i.e. those who would have been picked up through standard care).

All patients who did not opt out of HIV screening underwent venesection for HIV testing using the Abbott Architect HIV Ag/Ab Combo assay. Any patient with a reactive HIV antibody test received a Western blot confirmation at the National HIV Reference Laboratory. Blood draws for the screening tests were taken together with other routine blood tests.

The combined cost of the HIV antibody and Western Blot test is S$16.80 for subsidised patients and S$21.70 for non-subsidised patients.

HIV screening test results were conveyed to patients by the treating medical team if available prior to discharge or at follow-up in the Infectious Diseases clinic if results were pending at time of discharge. All patients screened and/or confirmed positive were referred to the Infectious Diseases service for evaluation.

### Evaluation of Reasons for Opting In or Out of the VOS Programme

Reasons for opting-in and opting-out of the program were reviewed using an interviewer-administered structured questionnaire in a representative sample of all eligible patients admitted in January 2013. This interview was carried out by four HIV screening coordinators who were trained by an Infectious Disease physician to minimize interviewer bias. Questions were standardized and prompting was discouraged when administrating the questionnaire. Verbal consent was obtained prior to the interview and patients were assured that clinical care would not be compromised if they declined to participate. Patient identifiers were not collected to maintain anonymity.

### Other Data

Electronic health records in the form of standard admission databases maintained by the hospital administration and prospectively collected HIV VOS program specific databases managed by the hospital’s Epidemiology Unit were accessed.

### Statistical methods

Analysis was conducted using SPSS software v.20 (IBM Corp., Armonk, NY). Statistical tests were two-sided, and p-value of <0.05 were considered to indicate statistical significance. Categorical variables were compared using Pearson Chi-square or trend Chi-square. ANOVA and Kruskal-Wallistest were used for parametric and non-parametric continuous variables respectively.

### Ethical approval

Ethics approval for the study was granted by the Domain Specific Review Board of Singapore’s National Healthcare Group. Informed consent was not obtained from participants for use of clinical data as data was anonymised, with no identifiers made available to researchers.

## Results

Between 1 January 2010 and 31 December 2013, there were 231,227 admissions to NUH. 107,523 (50.4%) were eligible for the HIV VOS program. 5215 (4.9%) of those eligible agreed to testing (opted-in). 4850 (93.1%) of those who opted-in had the HIV screening test done. Three (0.06%) patients of those who underwent the test tested positive for HIV.

Evaluation of the VOS programme using interviewer-administered questionnaires was conducted over one month from 17 Dec 12 to 11 Jan 13. During this period, 5051 patients were admitted of whom 2758 (50.6%) were eligible for HIV VOS; 34 (1.3%) patients opted in.

The HIV coordinators approached 478 patients, and 238 patients consented to the study (49.8% response rate, or 8.6% of total patients that were eligible for the VOS programme); of whom 21 (8.8%) opted in and 217 (91.2%) opted out. 14 of the 21 (66.7%) who opted in had the test performed. The results are summarised in Table 1.

**Table 1:** HIV VOS Testing Data and Survey Response Rate.

HIV VOS Testing Data from 1 Jan 10 to 31 Dec 13:	
Total Admissions	231,227
Number Eligible for HIV VOS	107,523 (50.4% of total admissions)
Opted in	5,215 (4.9% of eligible)
Number opted in tested for HIV	4,850 (93.1% of opt ins)
HIV Test Positive	3 (0.06% of those with test performed)
HIV VOS Testing Data and Survey Response from 17 Dec 12 to 11 Jan 13	
Total Admissions	5,051
Number Eligible for HIV VOS	2,758 (50.6% of total admissions)
Opted in	34 (1.3% of those eligible)
Number of patients approached for survey	478 (8.6% of those eligible)
Number of patients who consented to survey	238 (49.8% of those approached)
Number surveyed who opted in	21 (8.8% of those who consented to survey)
Number opted in tested for HIV	14 (66.7% of those who opted in)

The median age of the surveyed patients was 65 years and 133 (55.9%) were male. 90 (37.8%) had attended primary school, 85 (35.7%) had attended secondary school, and 45 (18.9%) had received post-secondary education; 17 (7.1%) did not report their education status. The ethnic distribution was: Chinese 124 (52.1%), Malay 69 (29.0%), Indian 44 (18.5%) and Others 16 (6.7%). 63 (26.5%) stayed in 3-room and smaller flats, 130 (54.6%) stayed in 4 or 5-room flats, 8 (3.4%) stayed in private housing whereas 9 (3.8%) were from nursing homes. 122 (52.6%) suffered from chronic disease (only 218 (91.6%) responded to this question)

### Opting In versus Opting Out

21 (8.8%) patients opted-in and the majority were male (76.2%). 14 of 21 patients who opted in were tested; none were positive. Patients who opted-in were younger (p for trend = 0.03), more educated (p for trend<0.01) and reported having more regular sexual partners (p for trend = 0.01). Type of housing, number of casual sexual partners, sexual orientation, intravenous drug use, condom use and previous sexually transmitted disease were not associated with opting-in or out of VOS (Table 2).

**Table 2:** Demographics and Risk Factors for HIV, by Opt-in and Opt-Out Status.

Demographics or Risk Factor	Opt-in (*n*, %)	Opt-out (*n*, %)	P value
Age			
21–30	3 (11.5)	23 (88.5)	0.03†
31–40	4 (13.8)	25 (86.2)	
41–50	5 (14.3)	30 (85.7)	
51–60	5 (10.2)	44 (89.8)	
61–70	4 (8.3)	44 (91.7)	
71+	0 (0.0)	50 (100)	
Gender			
Male	16 (76.2)	122 (56.2)	0.08*
Female	5 (23.8)	95 (43.8)	
Education			
Primary and below	3 (15.0)	90 (42.9)	0.00†
Secondary	10 (50.0)	77 (36.7)	
Polytechnic or Junior College	4 (20.0)	29 (13.8)	
University	3 (15.0)	14 (6.7)	
Housing Type			
Nursing home	0 (0.0)	10 (4.1)	1.00†
Rental flat	2 (9.5)	9 (4.1)	
2 room public housing	2 (9.5)	6 (2.8)	
3 room public housing	6 (28.6)	35 (16.1)	
4 room public housing	9 (42.9)	101 (46.5)	
5 room public housing	1 (4.8)	12 (5.5)	
Private	0 (0.0)	8 (3.7)	
Ethnicity			
Chinese	6 (28.6)	112 (47.5)	0.06*
Indian	7 (33.3)	37 (15.7)	
Malay	8 (38.1)	52 (22.0)	
Others	0 (0.0)	14 (5.9)	
Any chronic illness			
Yes	10 (47.6)	116 (56.6)	0.38*
No	11 (52.4)	89 (43.4)	
Condom use			
Never	15 (75.0)	126 (83.4)	0.58†
Sometimes	4 (20.0)	21 (13.9)	
Everytime	1 (5.0)	4 (2.6)	
Past HIV test			
Cannot recall	1 (5.0)	2 (1.1)	0.40†
Never	15 (75.0)	152 (86.4)	
1 Year	0 (0.0)	4 (2.3)	
5 Years	4 (20.0)	18 (10.2)	
Number of regular sexual partners in lifetime			
0	1 (6.2)	26 (15.0)	0.01†
1	11 (68.8)	139 (80.3)	
2	3 (18.8)	6 (3.5)	
3 or more	1 (6.2)	2 (1.2)	
Number of casual sexual partners in lifetime			
0	18 (100.0)	165 (97.1)	0.51†
1	0 (0.0)	3 (1.8)	
2	0 (0.0)	1 (0.6)	
3 or more	0 (0.0)	1 (0.6)	
Past sexually transmitted infection			
Yes	0 (0.0)	1 (0.05)	0.73*
No	20 (100.0)	169 (99.5)	

† By trend chi square test;

* By Pearson’s chi square

### Reasons for Opting In and Opting Out

The most common reasons cited by patients for opting-out (Table 3) were: belief that they were at low risk of HIV (50.0%), being too old (26.3%), not understanding the rationale for the test (7.4%), cost (6.9%) and aversion to venepuncture (6.5%). The most common reason for opting-in (Table 4) was desire to know their HIV status (47.6%).

**Table 3:** Reasons for Opting Out (n = 217).

I am at low risk of HIV Infection	109 (50.0)	96 (44.0)
I am too old	57 (26.3)	148 (68.2)
I did not understand what the test was for	16 (7.4)	189 (87.1)
The test is too costly	15 (6.9)	190 (87.6)
I am afraid of blood taking	14 (6.5)	191 (88.0)
I have been tested recently	6 (2.8)	199 (91.7)
I am afraid of finding out my HIV status	4 (1.8)	201 (92.6)
I already know my HIV status	4 (1.8)	201 (92.6)
My family is around	1 (0.5)	204 (94.0)
I am worried that testing is not anonymous	0 (0.0)	205 (94.5)
I am afraid of losing my job if I test positive	0 (0.0)	205 (94.5)

* A total of 217 patients who opted out answered the questions above; percentage was calculated as a proportion of 217

**Table 4:** Reasons for Opting in (*n* = 21).

Question	Yes (*n*, %*)	No (*n*, %*)
I want to find out my HIV status	10 (47.8)	11 (52.4)
I do not know my HIV status	2 (9.5)	19 (90.5)
I am at risk of HIV Infection	1 (4.8)	20 (95.2)
I have not been tested recently / ever been tested	1 (4.8)	20 (95.2)
I am feeling unwell	0 (0.0)	21 (100.0)
My partner told me to get tested for HIV	0 (0.0)	21 (100.0)

* A total of 21 patients who opted in answered the questions above; percentage was calculated as a proportion of 21

### Other Screening Programmes

Apart from the NUH HIV VOS program there are other HIV screening programs in Singapore (Table 5). Nationally, the HIV VOS program has been implemented in all public hospitals and has reported a higher prevalence per 1000 tested as compared to NUH. The Action for Aids (AFA) screening program targets high-risk groups, offering screening at bars, clubs, saunas and other high-risk venues in the community, whereas the antenatal screening program targets low risk mothers during antenatal check ups.

**Table 5:** Comparison of Various HIV Screening Programmes *.

	Population Group	Total Screened	Number Positive	Prevalence (per 1000 tested)
Action for Aids [27–29]	High risk; 33.3–40.4% MSM	18190	368	20.2
NUH VOS Program	Low risk; Inpatients	4850	3	0.62
Singapore VOS Program [30]	Low risk; Inpatient	101,131	114	1.13
Singapore Antenatal Screening [30]	Low risk	43,304	27	0.62

* Results for years 2010 to 2012

In NUH, the number of patients newly diagnosed with HIV picked up on routine clinical care (diagnostic testing for HIV-related medical complications) was 103.

## Discussion

### Routine Screening

The consequences of a large prevalence of undiagnosed HIV and late diagnosis are twofold: higher transmission rates [8–10] and higher morbidity and mortality [11,12].

Acute HIV infection is often missed due to non-specific signs and symptoms [13,14] with 90% of patients who seek medical care during the acute phase of HIV infection missed [15]. Risk based screening also fails to pick up a substantial number of cases as many individuals do not declare risk or identify themselves as low risk irrespective of actual risk [16–18]. Routine screening allows for the diagnosis of HIV in patients who may otherwise be missed.

### Poor Uptake of Screening and Low Prevalence of HIV in the Tested Population

Given that the estimated seroprevalence of HIV in Singapore is 0.28% [6] and that the proportion of late stage diagnoses amongst new HIV diagnoses had increased from 50% in 1994 to 57% in 2009 [19], far higher than the 32% reported by the US CDC [20], there is a strong case for routine screening in Singapore.

However, our HIV screening program has been unsuccessful mainly due to the low uptake rate of 4.9% compared with uptake rates between 21% and 98% reported by other investigators [21,22]. In our cohort, 0.62 per 1000 tested patients were positive for HIV, compared with 1.13 per 1000 patients tested nationally in similar HIV VOS programs and 20.2 per 1000 tested in targeted screening programmes run by the Action for Aids Singapore (AFA) in the same time period (Table 5). In addition, a study in another public hospital in Singapore reported higher uptake (21%) and test positivity (1.8 per 1000 screened) in a similar screening program [21]. Possible explanations for the differences may include different exclusion criteria for screening (NUH’s criteria was stricter, and hence less patients were eligible for screening), not excluding cases picked up through standard care in reporting of the number of positive HIV cases (NUH excludes cases picked up through standard care) or differences in program administration (programs are run differently across the hospitals; for example some hire and train dedicated staff to perform HIV screening while others use existing staff such as ward nurses who may have minimal training or inadequate time). Another possible reason is that internationally for most HIV VOS programmes, opt-out screening programmes entail informing patients that the screening test will be performed unless they object. No deliberate consent or counselling is recommended. However, in NUH, verbal consent is required prior to testing, which may reduce the uptake rate.

### Opting In

In our study, patients who opted in for testing were more likely to be male, have higher education and have had more regular sex partners over their lifetime. These results are consistent with other studies reporting an association between screening uptake with higher education [23] and male gender [21,24]. Some investigators have found a positive association between test uptake and of higher age [25] and female gender [23]. This variation could be due to underlying sociocultural and health policy and system factors.

Having more sexual partners increases the risk of acquiring HIV, and if patients recognize the increased risk, they may be more likely to take up screening. However, having a higher number of casual sexual partners was not associated with opting-in in this study, unlike what was observed in Hong Kong [25]. Sexual orientation and lifestyle did not but heightened awareness inferred by a higher education did influence patients to opt-into the screening program in our cohort.

The most cited reason for uptake of screening was the desire to find out one’s HIV status. It had been shown by Merchant et al that those that had previously undergone HIV screening because they wanted to find out their HIV status, were more likely to opt-in for HIV screening when offered than those who previously screened for other reasons [24].

### Opting Out

Self-perceived low risk of HIV is the top reason for declining HIV testing, similar to another local study [21]. However, self-perceived low risk may not reflect true low risk behaviour, but may instead be a function of poor understanding of the disease and risk factors [16,18,26]. There is some evidence that this may be the case in Singapore. In a targeted screening programmes by AFA in 2012, 12% of individuals who reported low risk behaviour, and 30% of those who reported that they were unsure of their risk profile tested positive for HIV under the programme [27]. Hence, while risk based screening remains a valuable tool, it is evident that it cannot be the only strategy.

In our study population, a significant number reported that they opted-out because they felt they were too old. This perception could be one reason why in Singapore, amongst those aged 55 years and above, late stage HIV diagnosis was 5 times more likely [19].

While cost, fear of venepuncture and poor understanding of the VOS programme were the other reported barriers, these could be easily overcome through increased subsidies, utilization of oral test kits and education aids to improve understanding. Fear of finding out their HIV status, social repercussions from a positive finding and patient confidentiality were notably not important factors for opting-out, unlike in other studies elsewhere [17,18,26].

### Limitations

The study did not examine the reasons for poor uptake of screening using established behavioural science theorems, which may have allowed for a more comprehensive assessment. Given the low number of patients who opted-in, the reasons for opting-in cannot be adequately evaluated. Interviewing a larger cohort would have allowed a better interpretation of the reasons for opting-in (a larger cohort was not interviewed in this study due to resource constraints; only a representative subset of eligible patients were surveyed).

## Conclusion

The success of a HIV VOS program is largely determined by test uptake. Patient education and empowerment to better understanding of the disease process, transmission risks, available treatment options and medical support systems to manage the disease once diagnosed, would better equip patients in self-risk assessment and decision to opt for the test. Systems level changes such as a more streamlined opt-out process that misses less eligible patients and more clearly conforms to the opt-out principle may help increase screening rates. However, a more important and challenging aspect that would greatly facilitate HIV test uptake, is the creation of an environment that stigmatizes HIV infection less. Until these factors are addressed, it is unlikely that programs such as ours will narrow the gap between known and estimated seroprevalence of HIV infection.
